# More of the same or a change of scenery: an observational study of variety and frequency of physical activity in British children

**DOI:** 10.1186/1471-2458-13-761

**Published:** 2013-08-16

**Authors:** Hannah L Brooke, Kirsten Corder, Simon J Griffin, Ulf Ekelund, Esther MF van Sluijs

**Affiliations:** 1 UKCRC Centre for Diet and Activity Research, Institute of Public Health, University of Cambridge, Cambridge, UK; 2 MRC Epidemiology Unit, University of Cambridge, Cambridge, UK; 3 Primary Care Unit, Institute of Public Health, University of Cambridge, Cambridge, UK; 4 Department of Sports Medicine, Norwegian School of Sports Sciences, PO Box 4014, Oslo, Ullevål Stadion 0806, Norway

**Keywords:** Children, Physical activity, Accelerometer, Primary school, Intervention, Public health

## Abstract

**Background:**

Physical activity is important for children’s health, but successful physical activity promotion is challenging. Whether performing many different types of activities (Variety) is associated with higher physical activity independent of the number of activity sessions (Frequency) is unknown, but this information could inform physical activity promotion and public health strategies in children.

**Methods:**

In the SPEEDY study we measured moderate-to-vigorous intensity physical activity (MVPA; ≥2000 counts/minute) over 7 days using GT1M Actigraph accelerometers in 1700 children from Norfolk, UK (56% girls, Mean ± SD 10.3 ± 0.3 years-old). Children reported participation in 28 leisure-time activities over the previous 7 days. Sex differences in activity participation were assessed using multilevel logistic regression, clustered by school. Associations of log-transformed MVPA with z-score-Variety (number of different activities/week) and z-score-Frequency (sum of all activity sessions/week) were examined using multilevel linear regression, adjusted for age, sex, parental education and age-standardised BMI.

**Results:**

Children’s activity participation often reflected gender stereotypes. Mean ± SD Variety was 10.8 ± 5.0 activities/week, and Frequency was 24.2±15.0 sessions/week. In separate models lnMVPA had similar strength, positive associations with z-score-Variety and z-score-Frequency (Exp β(95% CI); Variety 1.04(1.02-1.06), Frequency 1.04(1.02-1.06)). lnMVPA was not associated with z-score-Variety independent of z-score-Frequency (Variety 1.01(0.98-1.04), Frequency 1.03(1.00-1.06)).

**Conclusions:**

Future physical activity interventions and public health strategies could allow for gender specific activity preferences and could target both Variety and Frequency of activities participated in by children.

## Background

In children and adolescents, physical activity is inversely associated with metabolic syndrome and clustered cardiovascular risk factors [1-3]. The British government recommends that children participate in a minimum of 60 minutes of moderate-to-vigorous intensity physical activity (MVPA) each day [4], however, only 30–40% of youth (2–18 years old) meet these guidelines [5]. It is therefore important to develop effective interventions and public health strategies to promote physical activity in young people, particularly as interventions to date have had limited success [6].

Interventions to promote physical activity often aim to increase participation in specific modes of activity (i.e. specific activities, e.g. walking or dancing) but relatively little is known about the mode of activities participated in by British children and how this is related to objectively measured physical activity. There is some evidence regarding participation in specific activities among young people from Australia, New Zealand and USA but activity mode is likely to substantially differ across different countries due to both climate and cultural norms [7-11]. A study in British adolescents broadly grouped activities into six categories (housework, sedentary activities, outside activities, active jobs, sports participation and active travel), and suggested that activity type and context may vary according to objectively measured activity level [12].

The volume of activity recommended by physical activity guidelines can be achieved by performing few activities several times per week or by performing many different activities less often. However, it is unknown whether the number of types of activities children participate in each week (Variety) is associated with objectively measured physical activity independent of how often activity is performed each week (Frequency). Previous evidence is inconclusive indicating that although participation in a number of different physical activities may be important for activity maintenance throughout adolescence [11], proxy reported Variety was not associated with objectively measured physical activity in children and was not examined independent of overall activity frequency [13]. Therefore it is uncertain whether public health strategies and interventions to promote physical activity in children could benefit from specifically focusing on Variety or Frequency of activity participation or whether both are equally important.

It may be prudent for interventions promoting physical activity to be targeted towards particular population subgroups, such as girls or overweight individuals [14]. Understanding the characteristics of physical activity behaviour within subgroups and how it is associated with overall physical activity level could inform future physical activity guidelines and the development of effective strategies to promote physical activity. It could be beneficial for physical activity guidelines to recommend a minimum number of different types of activity for children to participate in, or to design an intervention with opportunities for children to try a wide range of different activities. These strategies may be more valuable for certain population subgroups than others.

We aimed to describe 10-year old children’s participation in common activities and examine differences in Variety and Frequency of activity participation between population subgroups. We also aimed to explore associations of self-reported Variety and Frequency of activities of at least moderate intensity with objectively measured physical activity, specifically focusing on MVPA as suggested by the current British physical activity guidelines [4].

## Methods

### Study outline

A cross-sectional examination of data from the Sport, Physical activity and Eating behaviour: Environmental Determinants in Young People (SPEEDY) study was conducted. Full ethical approval was given by the University of East Anglia local research ethics committee. Recruitment and methodology have been described previously [15]. Briefly, 157 out of 227 (69%) primary schools in Norfolk, Eastern England, with more than 12 pupils in Year–5 (aged 9 to 10 years) were approached. Ninety-two schools and 2064 pupils (57% of eligible sample) agreed to participate and were visited for measurements from April to July 2007. Written informed consent was obtained from all pupils (signed by a parent/guardian and the child).

Trained research assistants visited each school; they performed anthropometric measurements according to standard procedures and supervised the children completing questionnaires. Children were fitted with an accelerometer and instructed how to wear it. Each child took home a questionnaire for their parent or guardian to complete; they were asked to return this and their accelerometer to school eight days after the measurement day.

### Youth physical activity questionnaire (YPAQ)

Self-reported physical activity was assessed using the YPAQ. The YPAQ was based on the Children’s Leisure Activities Study Survey (CLASS) [16], but adapted for the British population and to capture activities in the last seven days rather than a ‘typical week’. The YPAQ has been shown to adequately rank individual’s overall physical activity in 12–13 year old children (n = 25) [17]. Children reported their participation in 28 moderate-to-vigorous intensity activities during non-school time over the last seven days. Each activity had four response categories (“never”, “one day”, “2 to 3 days” and “four or more”); these were recoded as 0, 1, 2.5 and 4.5 sessions/week for analysis.

Variety was calculated as the sum of types of activities reported to be participated in. Frequency was calculated as the summed sessions/week of all reported activities. Variety and Frequency were transformed to z-scores, calculated as (x – $\bar{\times }$/SD $\bar{\times }$) to allow comparison of β-coefficients across exposures.

Children were excluded from analyses if they responded to less than a third of YPAQ questions (n = 45). A further 202 children (12% of the sample), had incomplete YPAQ data; we assumed that these children did not participate in activities with no response. There were no substantial differences in results when sensitivity analyses excluding children with any missing YPAQ data were conducted.

### Accelerometry

Free-living physical activity was assessed over seven days with an ActiGraph accelerometer (GT1M, Actigraph LCC, Pensacola, US) [18]. Uniaxial accelerometers explain between 33 and 78% of the variance in energy expenditure derived from doubly labelled water and indirect calorimetry [19-21]. The children were asked to wear the accelerometer on an elastic waistband on their right hip during waking hours, except whilst bathing, showering or swimming [18]. Data were stored at 5-second intervals [22-25] and a custom programme (MAHUffe, http://www.mrc-epid.cam.ac.uk) was used for data reduction. Data processing was comparable with other studies of similar aged children [26-28]; the first day of data collection (partial day of measurement at school), periods of time when zero counts were recorded continuously for ten or more minutes (‘non wear time’) and counts recorded before 6 am and after 11 pm each day were removed. Children were excluded from analyses if valid accelerometer data were not obtained for at least 500 minutes per day on at least three days (n = 106 without three full measurement days and n = 90 with completely missing accelerometer data). Monitoring physical activity for 480 minutes for three days has achieved an intraclass correlation coefficient (inter-individual variation/total variation) of 0.70 in children of a similar age [28,29].

MVPA was defined as time spent at ≥2,000 counts per minute (cpm) each day and sedentary time was defined as time spent at ≤100 counts per minute (cpm) each day; both are presented as a daily mean value. A threshold of ≥2,000 cpm is equivalent to walking at approximately 4 km/h in children [30], and has been used previously to study associations between physical activity intensity and metabolic outcomes in children [31]. Preliminary analyses showed that MVPA did not have normally distributed residuals and so it was log-transformed before further analyses (lnMVPA). Accelerometer data were used to categorise children into those meeting and not meeting the current physical activity guidelines of at least 60 minutes MVPA each day [4]. The least and most active 10% of the sample (both n = 170) were determined based on minutes spent in MVPA.

### Measurement of covariates

Children self-reported their date of birth and age was calculated from the measurement date. Simple non-invasive anthropometry measures were conducted with children dressed in light clothing. Portable Leicester height measures were used to measure height to the nearest 0.1 cm. A non-segmental bio-impedance scale (Tanita, type TBF-300A) was used to measure weight to the nearest 0.1 kg. Body mass index (BMI) was calculated and age standardised BMI was derived [32]. Weight status was determined using sex- and age-dependent BMI cut-points (non-overweight vs overweight/obese) [32].

The parent or guardian self-reported their highest educational qualifications as a proxy measure of socioeconomic status, and home address was used to determine home location. Parent/guardian education level was grouped into ‘General Certificate of Secondary Education (GCSE) or lower’, ‘A-level or lower vocational training’ and ‘University or higher vocational training’. Children’s home location was classified as ‘rural’ (villages, hamlets and isolated dwellings), ‘suburban’ (town and fringe areas) and ‘urban’ (town/city with >10,000 inhabitants) [33]. Children were excluded from analyses if they had missing data for age (n = 0), sex (n = 0), BMI (n = 12) or parent/guardian education (n = 182).

### Final sample

Complete data for outcome, exposures and covariates were available for 1700 children. Children who were excluded from analyses were similar to children who were included for age, sex, BMI, minutes spent in MVPA and parent/guardian education. However, excluded participants spent less time sedentary (mean difference in sedentary time, 11.9 minutes/day, p-value = 0.032) and were more likely to live in an urban vs rural home location (p-value = 0.023) compared to those included in the final sample.

### Statistical analyses

In all statistical analyses ‘school’ was used as a clustering variable to take into account the sampling frame. Linear regression was used where the outcome variable was continuous and logistic regression was used when the outcome variable was categorical.

Multilevel regression analyses were used to test sex differences in descriptive data, sex differences in participation in each YPAQ activity and differences between subgroups in self-reported Variety and Frequency (sex, home location, weight status, meeting physical activity guidelines and parent/guardian education level).

The associations of Variety and Frequency with lnMVPA were also tested using multilevel regression. Variety and Frequency were first considered as exposures in separate models and then as exposures in the same model. Multicollinearity of Variety and Frequency was checked but was not deemed likely to influence the results (variance inflation factor; VIF = 3.77), despite strong correlation (r = 0.86) [34]. Models were adjusted for potential confounding variables: age, sex, parent/guardian education level and BMI z-score. Sensitivity analyses were conducted excluding children with any missing YPAQ data. If Variety or Frequency differed between strata of sample subgroups, interactions between the subgroup and Variety or Frequency were explored in associations with lnMVPA.

## Results

There were no sex differences for age, height or parent/guardian education level (Table 1). However, girls had higher weight, BMI, sedentary time, and were more likely to live in a suburban location compared with a rural location than boys. Boys had higher MVPA than girls and were more likely to be meeting activity guidelines.

**Table 1:** Descriptive characteristics of the sample of children from the SPEEDY study used in these analyses

	**Total (n = 1700)**	**Girls (n = 953)**	**Boys (n = 747)**	**P-value**
**Age (years)**	10.3 ± 0.3	10.3 ± 0.3	10.2 ± 0.3	0.282
**Height (cm)**	140.6 ± 6.7	140.7 ± 6.7	140.5 ± 6.6	0.587
**Weight (kg)**	36.6 ± 8.4	37.2 ± 8.8	35.8 ± 7.6	<0.001
**BMI (kg/m**^**2**^**)**	18.2 ± 3.1	18.5 ± 3.4	17.9 ± 2.8	<0.001
**MVPA (mins/day)**	73.8 ± 24.6	66.0 ± 20.8	83.8 ± 25.5	<0.001
**Sedentary time (mins/day)**	456.9 ± 53.9	461.7 ± 51.7	450.8 ± 56.2	<0.001
**Weight status**				
Non-overweight	1308 (77)	707 (74)	601 (80)	Ref
Overweight/obese	392 (23)	246 (26)	146 (20)	0.001
**Activity status**				
Not meeting government PA guidelines	526 (31)	387 (41)	139 (19)	Ref
Meeting government PA guidelines	1174 (69)	566 (59)	608 (81)	<0.001
**Parent/guardian education level**				
GCSE or lower	649 (38)	383 (40)	266 (36)	Ref
A-level or lower vocational	707 (42)	388 (41)	319 (43)	0.116
University or higher vocational	344 (20)	182 (19)	162 (22)	0.075
**Location†**				
Rural	557 (33)	293 (31)	264 (36)	Ref
Suburban	489 (29)	292 (31)	197 (27)	0.029
Urban	623 (37)	351 (38)	272 (37)	0.274

Data are presented as mean ± standard deviation and n (%) for categorical variables.

Ref is the reference category.

Differences between Girls and Boys (p-value presented in Table 1) were determined using multilevel linear or logistic regression depending on whether the dependent variable was continuous or categorical.

†31 participants included in the main analysis had missing data for location, the descriptive characteristics presented in Table 1 for location are based on the 1669 participants (936 girls, 733 boys) with data available.

*SPEEDY* Sports, Physical activity and Eating behaviour: Environmental Determinants in Young people, *BMI* body mass index, *MVPA* moderate-to-vigorous physical activity, *PA* physical activity, *GCSE* General certificate of secondary education.

**Figure 1:**
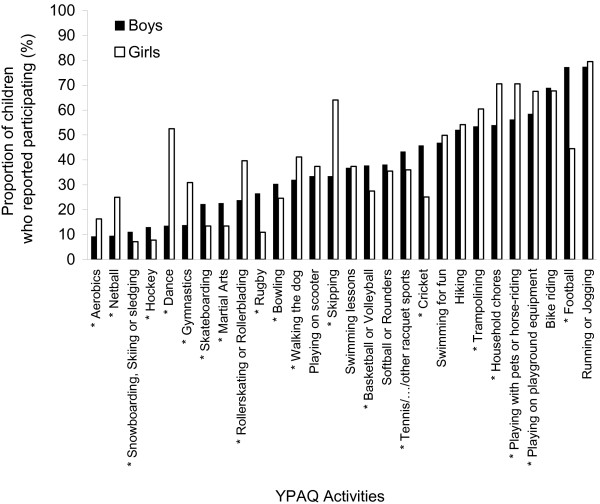
**Proportion of boys and girls reporting participating in each YPAQ activity.** * p < 0.05 for sex difference in the proportion of children participating in each activity. Tested using multilevel logistic regression. YPAQ; youth physical activity questionnaire.

Children reported participating in all 28 YPAQ activities; the proportion of children participating in specific activities ranged from 9% for ‘skiing, snowboarding, or sledging’ to 79% for ‘Running or jogging’ (Figure 1). Sex differences were observed for participation in 21 activities (75%); the largest sex differences in participation were seen for ‘Dancing’ and ‘Football’ with higher participation for girls and boys respectively (Figure 1).

Children participated in mean ± SD 10.8 ± 5.0 different types of activity each week (Variety) and in 24.2 ± 15.0 sessions each week (Frequency), these were both significantly (p <0.003) higher in the most active compared to the least active 10% of children (Table 2). Girls reported a higher Variety of activities than boys, and children who met physical activity guidelines reported a higher Variety and higher Frequency of activities than those who did not meet physical activity guidelines. In addition, children whose parent/guardian was in the highest educational group reported participating in activities less frequently than children whose parent/guardian had a low educational level. Therefore sex, meeting physical activity guidelines and parent/guardian education were taken forwards as potential moderating factors.

**Table 2:** Variety and Frequency of activities stratified by population subgroups

	**Variety**		**Frequency**	
	**Mean ± sd**	**P-value**	**Mean ± sd**	**P-value**
**Overall**	10.8 ± 5.0		24.2 ± 15.0	
Most vs. Least active*				
Bottom 10%	9.9 ± 4.7	Ref	21.0 ±12.7	Ref
Top 10%	11.7 ± 5.2	0.002	27.2 ± 14.8	<0.001
**Sex**				
Boys	10.4 ± 5.2	Ref	23.8 ±15.0	Ref
Girls	11.1 ± 4.9	0.011	24.6 ±15.0	0.299
**Location†**				
Rural	10.5 ± 4.7	Ref	23.4 ± 13.8	Ref
Suburban	11.1 ± 5.3	0.098	24.7 ± 15.6	0.155
Urban	10.8 ± 5.1	0.593	22.5 ± 14.0	0.413
**Weight status**				
Non-overweight	10.8 ± 5.1	Ref	24.1 ± 15.2	Ref
Overweight/obese	10.8 ± 4.6	0.893	24.6 ± 14.6	0.522
**Activity status**				
Not meeting government PA guidelines	10.4 ± 4.8	Ref	22.8 ± 14.4	Ref
Meeting government PA guidelines	11.0 ± 5.1	0.048	24.8 ± 15.3	0.009
**Parent/guardian education level**				
GCSE or lower	10.8 ± 5.0	Ref	24.5 ± 15.0	Ref
A-level or lower vocational	10.9 ± 5.2	0.710	24.8 ± 15.5	0.737
University degree or higher vocational	10.4 ± 4.7	0.210	22.5 ± 14.0	0.047

Data are presented as mean ± standard deviation.

Ref is the reference category.

Differences in Variety or Frequency between population strata (p–values presented in Table 2) were tested for using multilevel linear regression.

*comparison between the most (top 10% n = 170) and least (bottom 10% n = 170) active children based on number of minutes spent in MVPA.

†31 participants included in the main analysis had missing data for location, the descriptive characteristics presented in Table 2 for location are based on the 1669 participants (936 girls, 733 boys) with data available.

*PA* physical activity, *GCSE* general certificate of secondary education.

In separate models, Variety and Frequency had similar sized, significant, positive associations with lnMVPA, respectively accounting for 1.18 and 1.32% of the variance in objectively measured physical activity (Table 3). The effect size of Variety was smaller than that of Frequency and not significant when both were included as exposures in the same model. The results were similar when sensitivity analyses excluding all children with any missing YPAQ data were conducted. No significant interactions were identified, indicating that associations of Variety and Frequency with physical activity were similar for strata of population subgroups (Additional file 1: Table S1).

**Table 3:** Associations of objectively measured MVPA with self reported Variety and Frequency

		**Variety and Frequency in separate models**					**Variety and Frequency in the same model**				
**Outcome**	**Exposure**	**Exp. β**	**95% CI**		**P-value**	**% variance explained by exposure**	**Exp. β**	**95% CI**		**P-value**	**% variance explained by exposure**
MVPA	Variety	1.04	1.02	1.06	<0.001	1.18	1.01	0.98	1.04	0.402	1.36
	Frequency	1.04	1.02	1.06	<0.001	1.32	1.03	1.00	1.06	0.051	

Variety and Frequency were transformed to z-scores so effect sizes are directly comparable.

Associations were tested using multilevel linear regression.

Models were adjusted for age, sex, parental education level and age standardised body mass index (BMI).

MVPA; moderate-to-vigorous physical activity was log transformed for analysis. The β coefficients and 95% confidence intervals (95% CI) were then exponentiated for interpretation. Exponentiated β (Exp. β) and 95% CI represents a ratio of geometric means and can be interpreted in the same way as odds ratios. i.e. Exp. β and 95% CI <1 equates to a negative association whereas Exp. β and 95% CI >1 equates to a positive association.

P-value for significance of the association of Variety or Frequency with physical activity.

## Discussion and conclusion

Ten-year old children participated in a wide range of activities which often conformed to gender stereotypes. For example, a greater proportion of boys reported playing football compared to girls. This indicates that within physical activity promotion interventions and public health strategies it may be necessary to allow for personal and gender specific activity preference.

The most commonly reported activity was running, which contrasts with previous studies which have reported walking, basketball, swimming and football as the most common activities in young people [7,9-11,35]. Differences in activity participation from previous research may be due to heterogeneous cultures, age ranges, activity questionnaires and season in which data were collected. The current data were collected in spring and summer (April–July) and may reflect seasonal fluctuation in activity participation. For example, Rugby, which in Britain is typically played in winter, had lower participation than Cricket, which is traditionally played in summer. Furthermore, 9% of children reported participation in snow-based activities. Although there was no snow in Norfolk at the time of data collection there is a large ‘dry ski-slope’ in the county, which could explain this finding.

Differences in Variety and Frequency of activities between population subgroups (sex, activity status and parent/guardian education level) suggest that the characteristics of children’s physical activity behaviour are not homogenous. Tailoring interventions and public health strategies to the physical activity characteristics within the target population may therefore be appropriate. For example, an intervention targeting children who do not meet government physical activity guidelines may focus on increasing the Variety and Frequency of activities performed, as both are significantly lower than in children meeting the guidelines.

Results from separate models suggest that Variety and Frequency reflect objectively measured MVPA to a similar extent. However, the associations between Variety and MVPA were not independent of Frequency. This appears to suggest that if the total number of activity sessions performed is high, it may not matter whether children perform many activities few times per week or few activities many times per week. If both approaches result in high total Frequency of activity, they may both result in more MVPA overall. Therefore, both approaches could be relevant to health promotion depending on the population or personal preference. In addition, the lack of significant interactions indicates that the associations of Variety and Frequency with physical activity are similar across population subgroups. This suggests that Variety and Frequency may be relevant components of physical activity to promote for all children. The lack of independence between Variety and Frequency in associations with MVPA could be explained if Frequency is on the causal pathway between Variety and MVPA. It is possible that increasing Variety could increase Frequency and in turn MVPA. Therefore, offering a wide range of activities in interventions promoting physical activity or including a minimum recommended number of different activities in future physical activity guidelines may be relevant. Longitudinal data analysis or a randomized controlled trial is necessary to confirm or refute this suggestion as this cross-sectional study leaves potential for reverse causality.

### Strengths and limitations

It was possible to assess associations between objectively measured MVPA and two potentially important and mostly unexplored components of physical activity (Variety and Frequency). Moreover, data were obtained from a large population based sample, aiding the external validity of these results. However, the population of Norfolk is not representative of the rest of the UK [36], and results should be generalised to other populations with caution.

Accelerometers cannot accurately record activities without vertical hip movement e.g. cycling, and they must be removed for water based activities, therefore physical activity may have been underestimated in children who regularly cycle or swim. The focus of this paper was MVPA so sedentary time and light activity were not incorporated into the main models. The analyses could be repeated with objectively measured sedentary time and light activity as the main outcomes of interest as there may be different associations with Variety and Frequency of specific sedentary and light activities.

Self-report questionnaires are limited in their accuracy for estimating physical activity intensity and duration [5] but they can provide information about activity type that is not yet easily available from accelerometers. By examining self-reported Variety and Frequency, but not duration of activity the recall bias resulting from self-reported duration of activity should be minimised. We assumed that children’s ability to recall whether or not they participated in an activity may be more accurate than their recollection of activity duration. This is in line with the YPAQ being able to adequately rank children’s physical activity [17]. The questionnaire presented a list of pre-specified activity categories with multiple activities in some items such as ‘Basketball/volleyball’. Therefore, it is unavoidable that for some activities we lose detail about the specific activities that children participated in. Furthermore, the frequency of participation was reported in days per week with a maximum of ‘4 or more times’ in the last seven days. This may result in under-reporting of activities which could be participated in on multiple days and also multiple times per day. We assumed that the 202 children who had less than 1/3rd of YPAQ data missing did not participate in activities with no response. Although this could have led to some error, the sensitivity analysis conducted excluding children with any missing YPAQ data, indicated that this assumption did not affect the results.

Only a small amount of the variance in objectively measured physical activity could be accounted for by self-reported Variety and Frequency. This could be partly because self-reported and objective physical activity was not assessed simultaneously. In addition, the YPAQ only asked about non-school activities, whereas objectively measured physical activity was determined over the whole day. This approach was chosen to inform the development of interventions aiming to facilitate children to increase their activity level over the whole day.

Variety and Frequency were calculated such that when children reported an extra activity (i.e. increased their Variety by one) they also reported at least one extra session (i.e. Increased their Frequency by at least one). Data were tested for multicollinearity and results indicated that it was acceptable to include Variety and Frequency in the same model and therefore the observed associations are unlikely to be artefacts due to the study design [34].

### Future research

In future it may be possible to use novel technologies to objectively measure the types of activity being performed, allowing further exploration of the patterns and characteristics of physical activity mode to better inform the design and targeting of physical activity promotion strategies. Longitudinal research with objectively measured physical activity is also necessary to examine whether Variety and Frequency of activities in childhood are associated with activity maintenance, uptake and drop out throughout adolescence and adulthood [11].

### Conclusion and public health implications

Given the wide range of activities reported and the apparent sex differences in participation it may be beneficial for future interventions and public health strategies promoting physical activity in children to allow scope for personal and sex specific activity preference. Furthermore, tailoring interventions to the physical activity characteristics within a target population may increase the chance of developing intervention programs that are acceptable, thus facilitating uptake and adoption. There was no evidence in 9–10 year old British children that activity Variety was associated with objectively measured MVPA independent of activity Frequency. Increasing the Frequency of existing activity or introducing new types of activity may therefore both be potential physical activity promotion strategies. However, longitudinal research is needed to examine whether both aspects of activity are similarly associated with activity maintenance.

## Abbreviations

MVPA: Moderate-to-vigorous intensity physical activity; lnMVPA: Log-transformed moderate-to-vigorous intensity physical activity; SPEEDY: Sport, physical activity and eating behaviour: environmental determinants in young people; YPAQ: Youth physical activity questionnaire; BMI: Body mass index; VIF: Variance inflation factor.

## Competing interests

The authors declare that they have no competing interests.

## Authors’ contributions

Study conception, design and data collection were conducted by SG, UE and EvS. The research question was defined by HB, KC and EvS. HB performed data analyses. HB and KC drafted the manuscript. All authors provided critical input on the data analyses and all versions of the manuscript, all authors approved the final version.

## Pre-publication history

The pre-publication history for this paper can be accessed here:

http://www.biomedcentral.com/1471-2458/13/761/prepub

## Supplementary Material

Additional file 1: Table S1 Interactions between strata of population subgroups and Variety and Frequency in associations with objectively measured MVPA. Table of interactions between strata of population subgroups and Variety and Frequency in associations with objectively measured MVPA. Click here for file
