# Supplementary material for: More of the same or a change of scenery: an observational study of variety and frequency of physical activity in British children
Source: BMC Public Health. 2013 Aug 16;13:761. doi: 10.1186/1471-2458-13-761 (PMC3751669; doi:10.1186/1471-2458-13-761)
Supplement: Additional file 1: Table S1 — Interactions between strata of population subgroups and Variety and Frequency in associations with objectively measured MVPA. Table of interactions between strata of population subgroups and Variety and Frequency in associations with objectively measured MVPA. [file 1471-2458-13-761-S1.pdf]

Supplementary table 1. Interactions between strata of population subgroups and Variety and Frequency in associations with objectively measured MVPA

		Frequency				Variety			
		β Coef.	95% CI		p - value	β Coef.	95% CI		p - value
MVPA	**Sex** (boys = 0)	0.013	-0.016	0.042	0.374	-0.003	-0.032	0.026	0.825
	**Activity status** (Not meeting government PA guidelines = 0)	0.002	-0.019	0.022	0.851	0.002	-0.019	0.023	0.851
	**Parent/guardian education level** (GCSE or lower=0)								
	A-level or lower vocational	0.006	-0.021	0.033	0.656	0.018	-0.012	0.049	0.230
	University degree or higher	0.025	-0.016	0.066	0.236	0.016	-0.022	0.055	0.402

Associations were tested using multilevel linear regression.

Variety and Frequency were transformed to z scores so effect sizes are directly comparable.

Moderate-to-vigorous intensity physical activity (MVPA) was log transformed for analyses

Models were adjusted for age, sex, parental education level and age-standardised BMI.

Beta Coeffeicient (B coef.) and 95% confidence intervals (95% CI) <0 indicates that the effect size of the comparison group is smaller than the effect size of reference group whereas B coef. and 95% CI >0 indicates that the effect size of the comparison group is large than the effect size of the reference group

P – value for significance of the effect size differing between the reference and comparison groups.

GCSE; General Certificate of Secondary Education
